# Septic complications of head and neck infections as emerging conditions in the post-COVID-19 era: an epidemiological study in a pediatric care center in Sicily

**DOI:** 10.1186/s13052-025-02148-8

**Published:** 2025-12-28

**Authors:** Daria La Cognata, Maria Carla Finocchiaro, Gian Luca Trobia, Alfio Alfonso Azzolina, Vita Antonella Di Stefano

**Affiliations:** 1 Postgraduate Training Program in Pediatrics, Department of Clinical and Experimental Medicine, University of Catania, Catania, Italy; 2 Pediatric Unit and Pediatric Department Emergency Room, “Cannizzaro Emergency Hospital”,, Catania, Italy; 3 Unit of Otorhinolaryngology, “Cannizzaro Emergency Hospital”,, Catania, Italy

**Keywords:** Head and neck abscesses, Otomastoiditis, Retropharyngeal abscesses

## Abstract

**Background:**

Head and neck area abscesses are severe bacterial infections that commonly arise as complications of viral upper respiratory tract infections in pediatric patients. These infections can affect various anatomical structures, including the tonsils, retropharyngeal spaces, paranasal sinuses, middle ear, and salivary glands. The most frequent clinical presentations include otomastoiditis, retropharyngeal abscesses, and periorbital cellulitis. Although traditionally considered rare in developed countries, in recent years, particularly in the post-COVID-19 (COronaVIrus Disease 19) pandemic period, we have observed a notable increase in these complications at our center.

**Methods:**

We conducted a retrospective, single-center epidemiological study on our cases of septic complications involving the head and neck region, comparing two three-year periods (from January to December): 2017–2019 and 2022–2024. Cases were classified into four categories: otomastoiditis, periorbital cellulitis, retropharyngeal abscesses, and other abscesses (including lateral cervical and cerebral abscesses). For each year the incidence rate of these complications was calculated in relation to the total number of hospital admissions. Statistical comparison was performed using the Chi-square test.

**Results:**

The comparison between the two three-year periods (2017–2019 vs. 2022–2024) revealed a dramatic increase in head and neck suppurative infections: 8 cases in 2017–2019 compared to 56 cases in 2022–2024. The incidence rose from 0.32% to 2.42%, a statistically significant difference (*p* < 0.01). The incidence rate peaked in 2024, reaching 3.3%. The most frequent complication was periorbital cellulitis, followed by otomastoiditis. Notably, in 2022, a case of cerebral abscess occurred, requiring transfer to intensive care.

**Conclusions:**

Our study highlights an unexpected increase in head and neck suppurative-inflammatory complications. While recent publications have documented a rise in pediatric respiratory infections in the post-COVID-19 era, specific studies addressing the surge in these conditions remain scarce. This trend may be linked to the so-called COVID-19 immunity gap and/or to emerging patterns of antimicrobial resistance. However, given the retrospective observational design of our study, establishing a causal link is not possible. Pending further scientific evidence, enhanced surveillance remains essential to promptly identify these conditions, given their rapid progression and high morbidity.

## Background

Septic complications of head and neck infections represent a serious consequence of acute viral upper respiratory tract infections in the pediatric population. The main clinical presentations include otomastoiditis, retropharyngeal abscess, peritonsillar and lateral cervical abscesses, periorbital cellulitis, suppurative lymphadenitis, and, albeit rarely, meningo-encephalic abscess complications. These conditions tend to progress rapidly and may result in severe, potentially life-threatening sequelae if not promptly recognized. For instance, undiagnosed otomastoiditis can evolve into meningitis or cerebral abscess [1], while untreated retropharyngeal abscesses may lead to mediastinitis or sudden airway obstruction [2].

One of the main challenges in managing these infections lies in their diagnosis. Early symptoms are often nonspecific and mimic those of common viral illnesses, frequently resulting in delays in both recognition and initiation of appropriate treatment [3].

Over the past decades, thanks to improvements in hygiene, widespread antibiotic use and vaccination, the incidence of these complications has significantly decreased, to the point that they are often regarded as “diseases of the past” in high-income countries [4].

However, despite being traditionally considered rare in developed nations, between 2022 and 2024, we observed in our pediatric care center in Catania a substantial shift in their epidemiology, with a marked resurgence in the number of cases in the post-COVID-19 era. This phenomenon, still underrepresented in both international [5, 6] and national literature, prompted us to conduct a retrospective study to assess the true extent of the issue. The COVID-19 pandemic in fact represented a dramatic event that significantly altered the epidemiology of infectious diseases. During this period, public health measures such as lockdowns, social distancing, widespread use of face masks, and school closures were implemented to limit the spread of SARS-CoV-2 (Severe Acute Respiratory Syndrome Coronavirus 2), causing substantial disruptions to the traditional epidemiological patterns of viral illnesses. Notably, during lockdowns, a marked reduction in cumulative cases was observed for several viruses, including Influenza Viruses, Respiratory Syncytial Virus, Human Metapneumovirus and Rhinoviruses [7]. Conversely, following the relaxation of these measures, these infections experienced a resurgence [8]. In light of these circumstances, we focused our investigation on the epidemiological trends of head and neck complications in pediatric patients to better understand the impact of COVID-19 pandemic on these specific illnesses.

Herein, we present a case series of emergent septic complications of head and neck infections observed at the Pediatric Unit and Pediatric Department Emergency Room of Emergency “Cannizzaro” Hospital in Catania, Sicily, and analyzed their incidence rate in the pre- and post-COVID-19 periods.

## Materials and methods

### Study design

We conducted a retrospective, observational, single-center study at the Pediatric Unit and Pediatric Department Emergency Room of Emergency “Cannizzaro” Hospital in Catania, Italy. We analyzed all the pediatric cases with suppurative head and neck complications recorded during the period from January 2017 to December 2024.

Patients were categorized into four main diagnostic groups:

1. Otomastoiditis.
2. Periorbital cellulitis.
3. Retropharyngeal and/or peritonsillar abscesses.
4. Other abscesses (including laterocervical abscesses, adenophlegmons and cerebral abscesses).

For each patient, the following data were collected:

1. Age, sex, vaccination status (regular or irregular according to the national immunization schedule) and relevant medical history.
2. Type of suppurative head and neck infection.
3. Pathogens identified through culture or rapid antigen tests.
4. Antibiotic therapy administered and need for surgical intervention.
5. Length of hospital stay and complications.

For each year, the number of hospitalized cases with or without head and neck suppurative complications was recorded. The cases were categorized into two temporal groups based on the COVID-19 pandemic timeline. Specifically, patients enrolled between 2017 and 2019 were classified as part of the pre-pandemic era (Group 1), while those from 2022 to 2024 were categorized as part of the post-pandemic era (Group 2). The incidence of patients with each abscess category (i.e., otomastoiditis, periorbital cellulitis, retropharyngeal and/or peritonsillar abscesses, other abscesses), as well as the cumulative incidence of head and neck suppurative complications, was calculated annually and for both three-year periods. Comparisons between the two three-year periods were conducted using the Chi-square test for categorical variables through a 2 × 2 contingency table reporting the number of patients with or without suppurative complications in the pre-pandemic or post pandemic, respectively. A p-value of < 0.05 was considered statistically significant. All statistical analyses were performed using Microsoft Excel, version 2403 (build 17425.20176).

### End points

#### Primary end point

The primary endpoint, also referred to as the true endpoint, was to assess changes in the incidence of suppurative head and neck complications in the pediatric population before and after the SARS-CoV-2 pandemic. We compared data from the three-year periods 2017–2019 and 2022–2024, collected at the Pediatric Unit and Pediatric Department Emergency Room of the Cannizzaro Hospital in Catania. We did not specifically consider the period 2020–2021 as, due to the presence of the lockdown during the SARS-CoV-2 pandemic, no cases of head and neck suppurative complications were documented.

#### Secondary end points

The secondary endpoints, also termed surrogate endpoints, aimed to:

- Identify the most frequent type of otorhinolaryngological suppurative abscess in children.
- Analyze the demographic characteristics of affected patients, with a focus on age, sex distribution and vaccine coverage.
- Evaluate the frequency of causative pathogens isolated through culture tests.
- Describe the clinical course of these complications, particularly treatments administered and the need for surgical intervention.
- Compare our findings with the existing literature to explore potential factors underlying the observed post-pandemic increase.

### Inclusion and exclusion criteria

#### Inclusion criteria

- Age ranging from > 1 month to < 13 years old.
- Diagnosis of suppurative head and neck infection, confirmed clinically and/or radiologically by contrast-enhanced CT (Computed Tomography) or MRI (Magnetic Resonance Imaging).

#### Exclusion criteria

- Patients with non-suppurative infections, hospitalized for other clinical conditions.
- Patients with chronic underlying conditions predisposing to infection (e.g., primary or acquired immunodeficiencies, malignancies).
- Patients with incomplete clinical information.

## Results

**Fig. 1:**
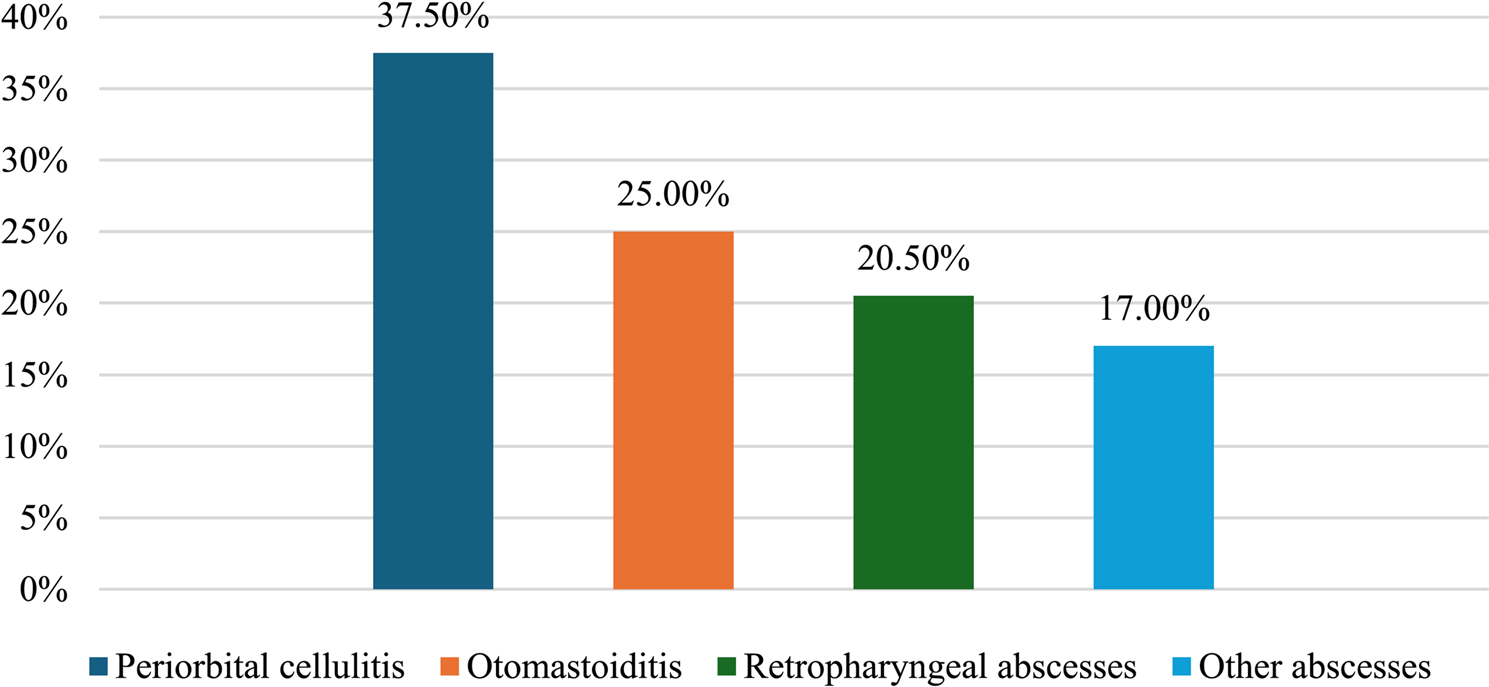
Subtypes of septic complications of head and neck infections. Pediatric Unit, “Cannizzaro” Hospital, Catania

Between 2017 and 2024 a total of 4.834 pediatric admissions were recorded at the Pediatric Unit and Pediatric Department Emergency Room of Emergency “Cannizzaro” Hospital in Catania. Among these, 64 cases involved suppurative complications of the head and neck region, corresponding to an overall incidence of 1.30%. Patients were categorized into four diagnostic groups: otomastoiditis, orbital cellulitis, retropharyngeal and/or peritonsillar abscess, and other abscesses (Fig. 1). Orbital cellulitis emerged as the most common complication, accounting for 24 of the 64 cases (37.5%), followed by otomastoiditis in 16 patients (25%). Retropharyngeal abscesses represented the third most frequent condition, with 13 cases (20.5%). The remaining 10 patients (15.5%) were diagnosed with other types of abscesses, including sinogenic abscesses and adenophlegmons. Notably, this group included one case (1.5%) of cerebral abscess, which developed as a severe complication of a head and neck suppurative infection.

**Fig. 2:**
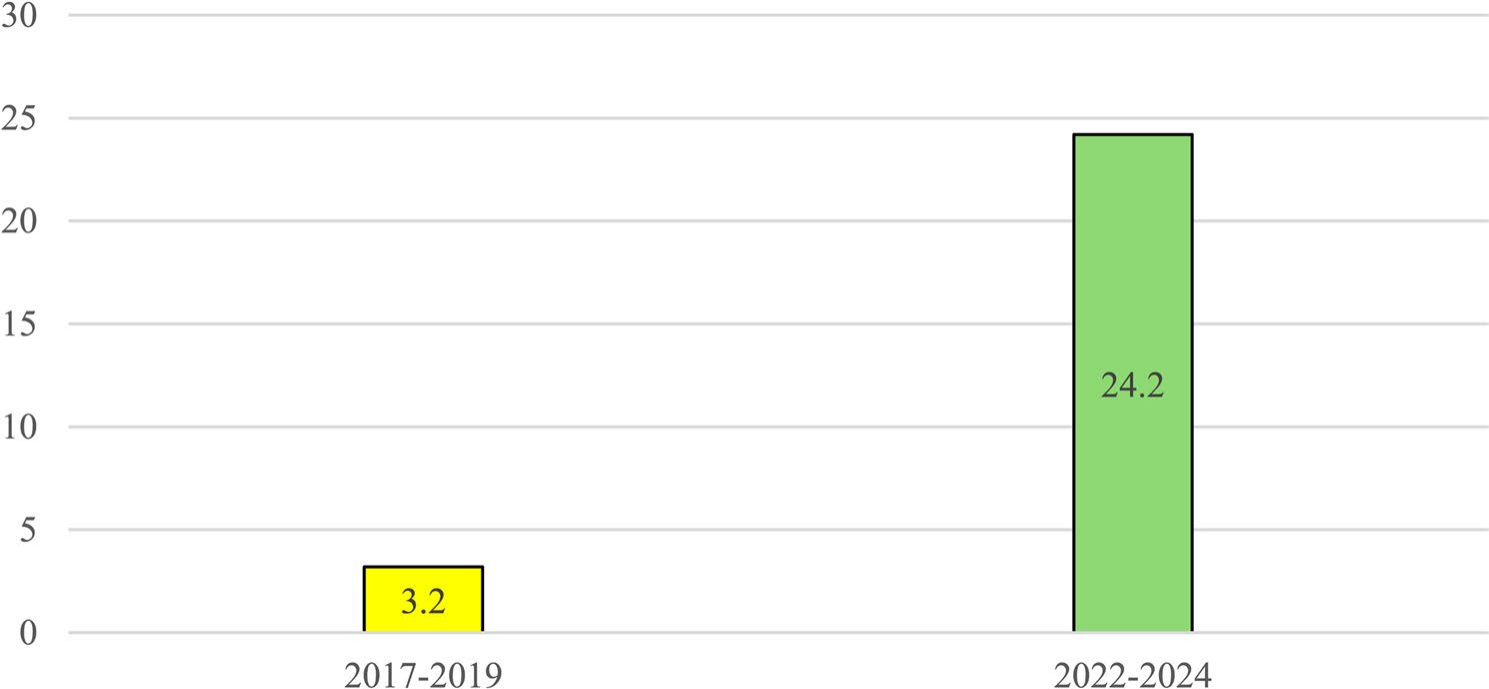
Number of cases of septic complications of ENT infections (per 1000 patients). Pediatric Unit, “Cannizzaro” Hospital, Catania

The temporal analysis revealed a significant increase (*p* < 0.05, Chi-square statistic 40.9532) in the incidence of head and neck suppurative complications in the 2022–2024 period, compared to 2017–2019. Specifically, while 8/2523 cases were recorded in 2017–2019 years with an average incidence of 0.32%, the 2022–2024 saw a marked rise to 56/2311 cases, corresponding to an average incidence of 2.42% (Fig. 2). Notably, no cases were documented in 2020 or 2021, coinciding with the lockdown period during the SARS-COV-2 pandemic.

**Fig. 3:**
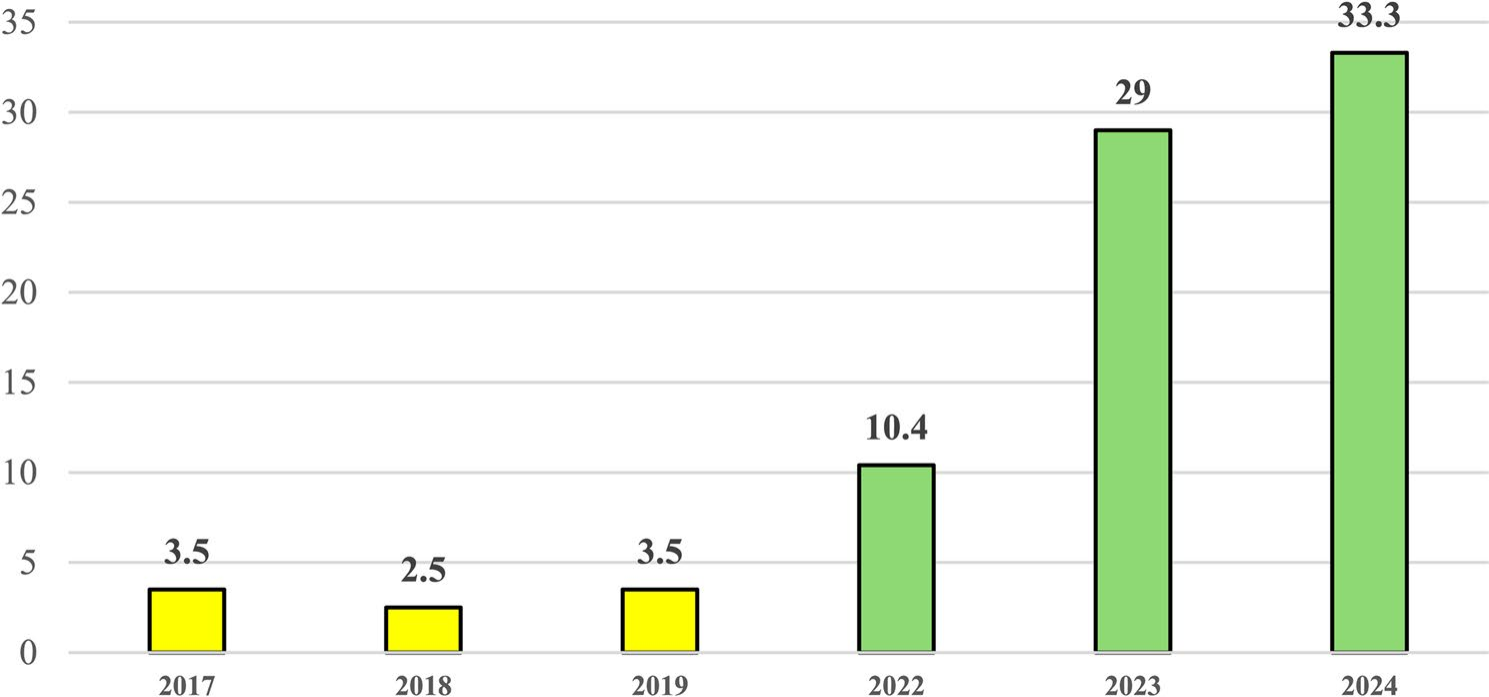
Incidence of septic complications of ENT infections (per 1000 patients). Pediatric Unit, “Cannizzaro” Hospital, Catania

Upon further analysis, during the 2017–2019 period, the incidence of these complications ranged between 0.25% and 0.35%. In 2022, this rate rose to 1.04%, followed by a further increase to 2.9% in 2023, peaking at 3.3% in 2024 (Fig. 3). A marked rise was observed during the first half of 2024, followed by a decline in the third quarter (from July 2024 to September 2024), and a slight resurgence in the final quarter (from October 2024 to December 2024).

**Fig. 4:**
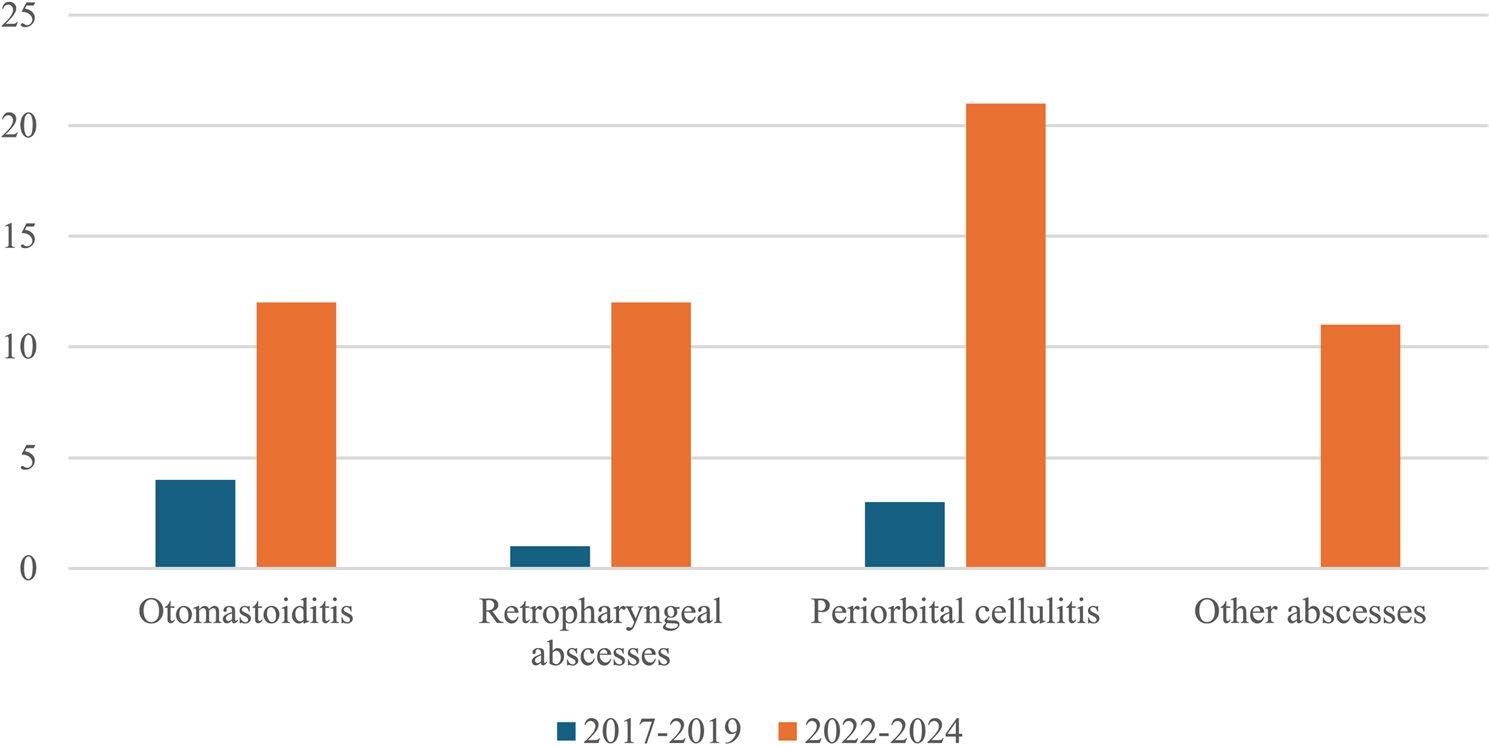
Subtypes of head and neck infections: 2017–2019 vs. 2022–2024. Pediatric Unit, “Cannizzaro” Hospital, Catania

A more detailed analysis of the individual subtypes revealed a consistent upward trend across all categories. Cases of otomastoiditis rose from 4 in the 2017–2019 period to 12 in 2022–2024. Retropharyngeal abscesses, which accounted for just one case in the earlier triennium, increased markedly to 12 cases in the more recent period. Periorbital cellulitis showed the most dramatic rise, from 3 cases to 21. Specifically, no cases of “other abscesses” (including lateral cervical abscesses, adenophlegmons, and intracranial complications) were observed in our department in the first triennium, whereas 11 such cases were documented between 2022 and 2024 (Fig. 4).

Period analysis revealed a clear increase in case numbers during the first two quarters of each year, with a higher concentration of infections occurring in the winter and spring months. This trend appears to correlate with the seasonal rise in upper respiratory tract infections typical of these periods.

**Fig. 5:**
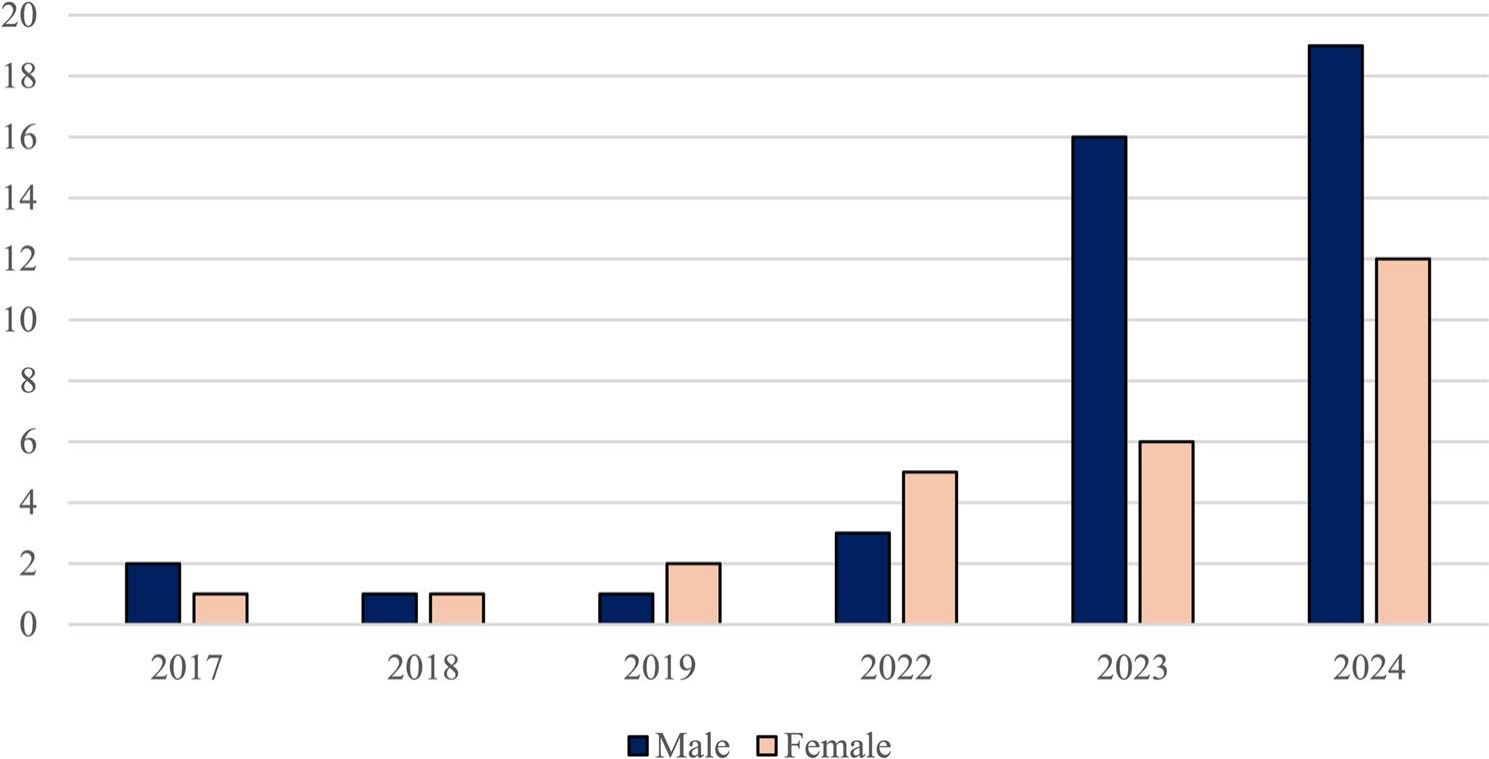
Head and neck infections: male vs. female. Pediatric Unit, “Cannizzaro” Hospital, Catania

Regarding sex and age distribution, males were more frequently affected than females, with a male-to-female ratio of 1.56:1 (39 males vs. 25 females) (Fig. 5). The overall mean age of affected children was 6.4 ± 3.7 years. In particular, a decrease in average age was observed in the post-pandemic triennium, from 7.6 ± 4 years in 2017–2019 to 6.1 ± 3.6 years in 2022–2024. When stratifying by age group, most cases (53%) occurred in children aged 0–5 years, followed by those aged 5–10 years (30%) and 10–13 years (17%). Overall, more than 70% of patients were under 6 years of age. Regarding vaccination coverage, half of the eight patients enrolled between 2017 and 2019 were compliant with the national immunization schedule, while the vaccination status of the remaining four was unknown. Among patients enrolled between 2022 and 2024, three were non-compliant and data were missing for 12 individuals. Given these limitations, comparison of vaccination coverage between the pre- and post-pandemic groups was not informative.

From an etiological standpoint, streptococcal and staphylococcal species emerged as the predominant bacterial pathogens responsible for severe head and neck infections. Specifically, in the 2017–2019 triennium, Streptococcus pyogenes was isolated in a single case via tonsillar swab, while blood cultures were negative in all patients during that period. In contrast, during the 2022–2024 triennium, microbiological investigations yielded more diverse results: two cases of Staphylococcus aureus and two of Streptococcus pyogenes were isolated from tonsillar swabs, Pseudomonas aeruginosa was detected in one auricular swab, and two cases of Streptococcus pneumoniae were identified via blood culture.

Although these infections follow their own bacterial epidemiology independent of viral pathogens, our data revealed a frequent association with either viral co-infections or secondary bacterial infections following viral upper respiratory illnesses. Due to technical limitations and inconsistent testing across cases, we were unable to quantify the exact prevalence of viral co-pathogens, as a nasal swab for respiratory viruses was not performed in all patients enrolled in the study. Nevertheless, based on available data, the most frequently detected virus in co-infections was Rhinovirus, followed by Influenza A and B viruses, Adenovirus, and Human Metapneumovirus.

As for diagnosis, contrast-enhanced CT remained the most reliable imaging modality for the diagnosis of deep neck infections and other suppurative complications in the head and neck region.

Regarding treatment, our experience supports the effectiveness of empiric antibiotic therapy with third-generation cephalosporins (e.g., ceftriaxone) in combination with clindamycin in complicated cases, defined as lack of clinical or laboratory improvement after 48 h and/or markedly elevated inflammatory markers at presentation.

Surgical drainage was necessary in 11 cases (17% of total), including 1 case in the pre-pandemic period and 10 in the post-pandemic period.

Although no statistically significant differences were found between the two periods in terms of clinical outcomes or length of hospital stay, a trend toward greater clinical severity was observed in the post-pandemic period. This included longer admissions and a higher rate of surgical intervention, suggesting a possible change in the natural course of these infections following the COVID-19 pandemic.

**Fig. 6:**
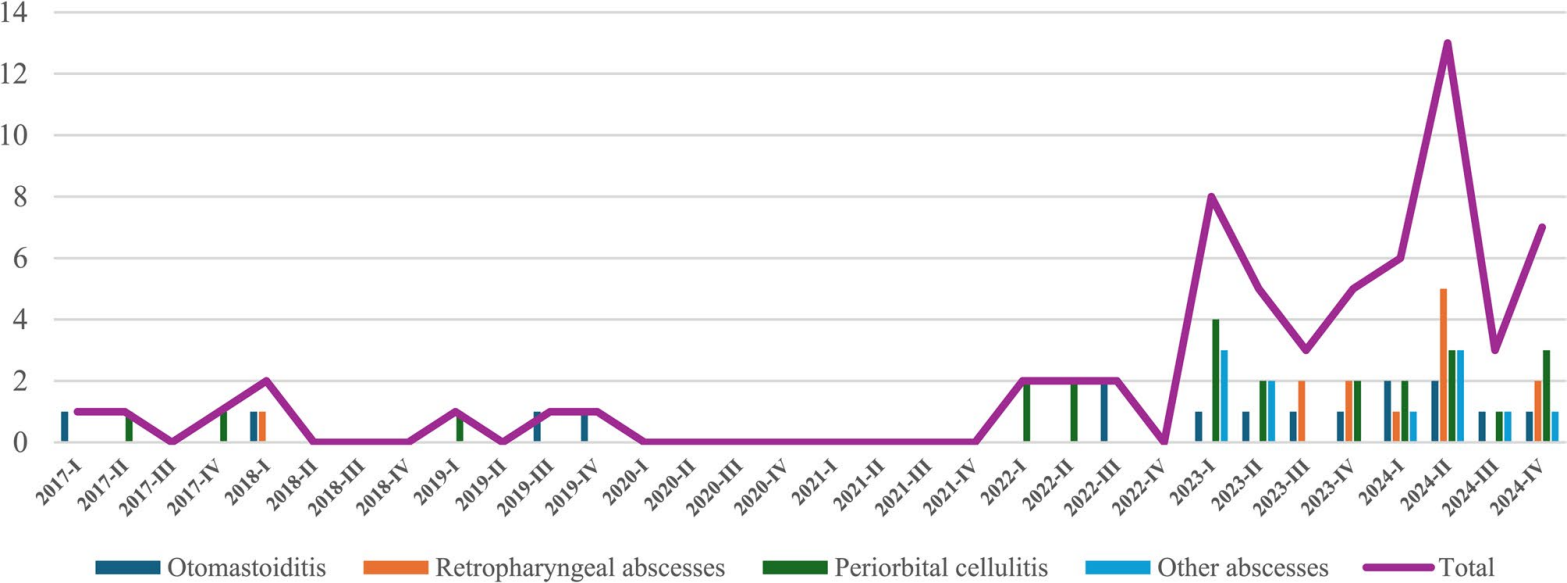
Epidemiology of septic complications of head and neck infections: 2017–2024. Pediatric Unit, “Cannizzaro” Hospital, Catania

Our analysis demonstrates a progressive and statistically significant rise in the incidence of complicated head and neck infections in the pediatric population, culminating in a peak during the first half of 2024 (Fig. 6). This upward trend has been paralleled by an increase in clinical severity, with a growing number of patients presenting with advanced suppurative complications. Of particular concern, a case of brain abscess was documented in 2022, necessitating transfer to the pediatric intensive care unit, highlighting the potentially life-threatening course these infections can take when diagnosis or treatment is delayed.

## Discussion

Our study highlighted a significant increase in head and neck suppurative complications in the pediatric population during the post COVID-19 pandemic period. This trend appears to be multifactorial, potentially attributable to several interacting factors, including the so-called immunity gap, increased antimicrobial resistance, changes in vaccine coverage, possible enhanced virulence of pathogens involved and a rise in respiratory viral infections acting as predisposing factors.

The immunity gap [9] (immunity gap or debt) refers to the reduced exposure of young children to common pathogens during the COVID-19 pandemic, due to public health measures such as lockdown, social distancing, widespread use of face masks and school closures. This limited exposure may have resulted in insufficient immune system training, leading to a greater vulnerability to severe bacterial infections upon resuming normal social interactions. Recent studies have already described this phenomenon in relation to other pediatric infections, including Respiratory Syncytial Virus (RSV) [10], Rhinovirus, Influenza A/B, and Streptococcus pyogenes [11, 12]. Our findings suggest that a similar mechanism may underlie the observed increase in head and neck suppurative complications during 2022–2024.

Another key factor identified in our study is the potential impact of increasing antimicrobial resistance. During the COVID-19 pandemic, antibiotic use was at times inappropriate, often being prescribed empirically, frequently without microbiological confirmation. This trend may have contributed to the selection of multidrug-resistant bacterial strains [13], reducing the effectiveness of first-line antibiotic therapies for upper respiratory tract infections. As a result, an increased risk of initial treatment failure and faster progression to severe and complicated clinical presentations, such as those observed in our cohort, may have occurred. Moreover, emerging evidence suggests increased virulence of certain pathogens in the post-pandemic period. For instance, variants of Streptococcus Pyogenes and Staphylococcus Aureus demonstrate enhanced ability to invade deep tissues, contributing to a rise in complicated infections. This could help account not only for the greater incidence observed, but also for the more severe clinical manifestations reported in recent years.

A particularly relevant aspect is the potential role of common viral infections as predisposing factors. Suppurative complications of head and neck infections are frequently the result of bacterial superinfections following otherwise mild viral upper respiratory tract infections. Recent literature has highlighted a post-pandemic surge in pediatric viral illnesses, with a notably aggressive re-emergence of respiratory pathogens such as RSV, Adenovirus, and Parainfluenza Viruses [14]. This viral rebound may have created a favorable background for secondary bacterial infections, thereby contributing to the increased incidence of abscess-related complications observed in recent years. Nevertheless, it remains to be determined whether this rise is solely attributable to a greater circulation of respiratory viruses or whether other concurrent factors, such as immune dysregulation or altered pathogen virulence, may have further predisposed to the development of severe bacterial complications.

Some authors have proposed that SARS-CoV-2 infection itself may contribute to a dysregulation of the immune system, leading to what has been termed “immunity theft” [15]. This theory suggests that the virus may impair the innate and adaptive immune responses, thereby reducing the host’s ability to mount an effective defense against subsequent bacterial infections. In pediatric patients, such immunological alterations could increase vulnerability to secondary invasive infections, including severe head and neck suppurative complications, and may partially explain the rise in both frequency and severity of these conditions in the post-pandemic period.

The resurgence of septic complications in the head and neck region during the post-pandemic period may be related, among other factors, to reduced paediatric vaccination coverage, possibly influenced by the lockdown. In our cohort, the lack of complete data did not allow us to determine whether variations in vaccination coverage contributed to the increase in cases or the younger age at onset. Further studies are needed to clarify the potential protective role of standard vaccinations in these events. 

Finally, another possible contributing factor may lie in long-term alterations of the airway microbiome, which could have predisposed children to more invasive diseases in the setting of viral infections. Changes in microbial colonization patterns following the pandemic period—due to reduced exposure, antibiotic use, or viral co-infections—might have disrupted the balance of commensal flora, thus lowering the natural defenses of the upper airways.

As for risk factors, our demographic analysis confirmed that male children and those under 5 years of age were the most affected. This increased vulnerability may be related to immunological differences between sexes, already described in other pediatric infectious diseases, and to the reduced ability of younger children to effectively manage upper respiratory tract infections, which often serve as the initial focus for suppurative complications.

The combination of these factors points to a multifactorial etiology (immunological, microbiological, and therapeutic) underlying the observed rise in complicated infections during the post-pandemic period.

Nevertheless, the specific role of viral infections in this trend remains an open question: was the resurgence of viral illnesses the primary trigger or did other factors, such as shifts in bacterial colonization patterns and altered immune responses, contribute equally to the increased incidence and severity of complications? Further research will be essential to clarify these dynamics and to provide targeted strategies for the prevention and early management of pediatric infections.

The number of international studies addressing this specific topic remains limited, and data at the national level are even scarcer. Our findings are consistent with those reported by Tsai et al. [5] who investigated the incidence of severe head and neck infections in children in the United States from 2018 to 2023. Their study documented a marked increase in such infections during the post-pandemic period. Notably, Tsai and colleagues observed a significant rise starting in 2021, with a 104% increase compared to the pre-pandemic period, which was attributed to a surge in both viral and bacterial infections following the relaxation of COVID-19 restrictions. Interestingly, while the United States experienced this rise as early as 2021, a similar trend emerged in our center with a delay of approximately one year. This temporal gap could be explained by the fact that pandemic containment measures in Italy (such as mandatory mask-wearing and social distancing) remained in effect longer than in the United States, with mask mandates extended until June 2022 [16]. Similarly, whereas Tsai et al. reported the peak incidence in 2023, our data indicate that the highest number of septic complications of head and neck area occurred in the first half of 2024. This phenomenon also appears to be specific to the pediatric population. In fact, Tsai et al. reported that the incidence of deep neck space infections among adults remained largely unchanged before and after the pandemic. Furthermore, their analysis of pediatric osteomyelitis cases [5] found no statistically significant variation in incidence across the same timeframes. Observations from European centers corroborate the trend we identified. Issa and colleagues [17], in a study conducted in Germany, reported a rise in brain abscesses as a complication of otomastoiditis during the post-pandemic period. This finding is consistent with the multicenter study by Massimi et al. [18], which involved several pediatric centers across Italy and documented a notable increase in complications arising from otitis media and sinusitis. Additional support comes from the work of Metz and colleagues [19], who also observed a higher incidence of complicated infections following the pandemic, and from Draut et al. [20], who, in another German cohort, reported an increase in pediatric otomastoiditis; their study highlighted the frequent isolation of Streptococcus Pyogenes and Streptococcus Pneumoniae, reinforcing the hypothesis of a shift toward more invasive bacterial infections in the pediatric population during the post-pandemic era. Similarly, VoB et al. [21] and Lohnherr et al. [22], both reporting from German centers, documented a noticeable increase in cases of orbital cellulitis and otomastoiditis during the post-pandemic period.

Consistently with our experience, several other centers have reported a notable increase in surgical interventions for complicated head and neck infections during the post-pandemic period. Hollborn et al. [23] observed a marked rise in the number of mastoidectomies performed after the pandemic on patients of all ages, suggesting a greater severity and progression of otologic infections. Similarly, Galli [24] documented a significant increase in surgically treated suppurative infections of the head and neck region on patients of all ages, accompanied by a higher rate of β-hemolytic streptococcal isolates.

Overall, our study confirms and expands upon existing international literature, highlighting a rising trend in pediatric head and neck suppurative infections in the post-pandemic era. This increase is likely multifactorial, driven by a complex interplay of epidemiological, microbiological, and immunological factors that warrant further investigation to be fully elucidated.

## Limitations and strengths

This study has some limitations:

- It is a single-center study, and therefore the sample size is relatively small.
- Complete data on vaccination coverage could not be collected, as this information was missing for a subset of patients.
- It was not possible to collect complete data on viral co-infections for all patients, as a nasal swab for respiratory viruses was not performed in all patients enrolled in the study.

However, the study also presents notable strengths:

- The findings are consistent with international literature and provide a valuable contribution to the national Italian perspective on this topic.

## Conclusion

Suppurative complications of head and neck infections in children have significantly increased in the post-pandemic period. This trend highlights the need for vigilant epidemiological surveillance and the implementation of targeted strategies aimed at the early prevention and management of pediatric infections, to prevent progression toward severe complications. The observed shift in epidemiological patterns underscores the pivotal role of the pediatrician in the early identification of infections, which is crucial to preventing serious complications. The rising incidence of these conditions also calls for a reassessment of current therapeutic approaches, with particular attention to the selection of appropriate antibiotic regimens and timely surgical intervention when needed. Finally, the integration of shared diagnostic and therapeutic protocols among pediatricians, ENT (Ear, Nose, Throat) specialists, and infectious disease experts is essential to optimize the management of complicated head and neck infections in children. Given the substantial impact of the COVID-19 pandemic on the pediatric infectious disease landscape, there is a clear need for continuous monitoring and a deeper understanding of the factors contributing to this epidemiological shift. Our study contributes to this growing body of evidence and aims to serve as a foundation for future multicenter research initiatives, with the goal of improving preventive and therapeutic strategies for head and neck suppurative infections in children.

## Data Availability

The datasets generated during and/or analyzed during the current study are available from the corresponding author upon reasonable request.

## Abbreviations

COVID-19: COronaVIrus Disease 19
SARS-CoV-2: Severe Acute Respiratory Syndrome Coronavirus 2
CT: Computed Tomography
MRI: Magnetic Resonance Imaging
RSV: Respiratory Syncytial Virus
ENT: Ear, Nose, Throat
